# Identification of Denatured Biological Tissues Based on Compressed Sensing and Improved Multiscale Dispersion Entropy during HIFU Treatment

**DOI:** 10.3390/e22090944

**Published:** 2020-08-27

**Authors:** Bei Liu, Runmin Wang, Ziqi Peng, Lingjie Qin

**Affiliations:** 1 College of Mathematics and Physics, Hunan University of Arts and Science, Changde 415000, China; liubei0112@smail.hunnu.edu.cn (B.L.); pengzq@huas.edu.cn (Z.P.); 2 College of Information Science and Engineering, Hunan Normal University, Changsha 410081, China; 3 School of Computer and Information Engineering, Central South University of Forestry and Technology, Changsha 410000, China; chqliu@huas.edu.cn

**Keywords:** HIFU, ultrasonic scattered echo signal, CS, IMDE, denatured tissue

## Abstract

Identification of denatured biological tissue is crucial to high-intensity focused ultrasound (HIFU) treatment, which can monitor HIFU treatment and improve treatment efficiency. In this paper, a novel method based on compressed sensing (CS) and improved multiscale dispersion entropy (IMDE) is proposed to evaluate the complexity of ultrasonic scattered echo signals during HIFU treatment. In the analysis of CS, the method of orthogonal matching pursuit (OMP) is employed to reconstruct the denoised signal. CS-OMP can denoise the ultrasonic scattered echo signal effectively. Comparing with traditional multiscale dispersion entropy (MDE), IMDE improves the coarse-grained process in the multiscale analysis, which improves the stability of MDE. In the analysis of simulated signals, the entropy value of the IMDE method has less fluctuation compared with MDE, indicating that the IMDE method has better stability. In addition, MDE and IMDE are applied to the 300 cases of ultrasonic scattered echo signals after denoising (including 150 cases of normal tissues and 150 cases of denatured tissues). The experimental results show that the MDE and IMDE values of denatured tissues are higher than normal tissues. Both the MDE and IMDE method can be used to identify whether biological tissue is denatured. However, the multiscale entropy curve of IMDE is smoother and more stable than MDE. The interclass distance of IMDE is greater than MDE, and the intraclass distance of IMDE is less than MDE at different scale factors. This indicates that IMDE can better distinguish normal tissues and denatured tissues to obtain more accurate clinical diagnosis during HIFU treatment.

## 1. Introduction

High-intensity focused ultrasound (HIFU) has strong penetrability and focusing capability. As a noninvasive modality for conducting high-temperature thermal therapy, HIFU can focus ultrasonic energy on a target region of the body to kill the cells in the treatment region [1]. In modern medicine, HIFU is often used to treat diseases such as cancer and tumors [2,3,4]. Overtreatment will cause normal tissue to be damaged, so it is important to monitor whether the biological tissues have been denatured in the HIFU treatment region [5]. HIFU treatment monitoring has three kinds of monitoring methods, including computed tomography (CT) [6,7], magnetic resonance imaging (MRI) [8,9], and ultrasound [10,11]. However, CT may damage the human body in HIFU monitoring, especially the elderly and children. MRI is also limited due to poor real-time performance, high price, and poor equipment magnetic compatibility. Ultrasound has become a research hotspot for HIFU treatment monitoring due to low cost, good real-time performance, and compatibility with hyperthermia devices [12,13]. For example, the ultrasound techniques of quantitative ultrasound, ultrasound elastography, and ultrasound entropy imaging are employed to monitor thermal ablations [14,15,16].

Ultrasonic signals contain a lot of noise during HIFU treatment, so we need to denoise the signals. However, traditional wavelet and empirical mode decomposition (EMD) ultrasonic denoising methods have some disadvantages. For example, the wavelet denoising method requires a preset wavelet basis function, and the EMD method is prone to modal aliasing problems during decomposition [17,18]. Thus, the CS method is used to denoise the ultrasonic echo signal during HIFU treatment [19]. In the process of CS analysis of the signal, CS can transform the signal from the time domain to other sparse domains. Then, the OMP method is used to reconstruct the signal to obtain the ultrasonic echo signal after denoising [20]. The CS-OMP method can obtain better denoising effects.

The ultrasonic echo signal is a nonlinear signal during HIFU treatment. Some properties of the ultrasonic echo signal change when the biological tissues have been denatured. Researchers in the field of ultrasound have studied the characteristics of ultrasonic attenuation, sound velocity, and entropy of ultrasonic echo signals, and hope to find characteristics that can accurately reflect the characteristics of biological tissues. In [21,22], the attenuation coefficient was used to evaluate thermal lesions. In [23,24], values of acoustic absorption and sound velocity were used to predict temperature rise and estimate treatment. In [25,26], biological tissue state after HIFU irradiation was assessed by information entropy of the radio frequency ultrasound. Comparing with ultrasonic attenuation, sound velocity, and information entropy, multiscale permutation entropy (MPE) can analyze sequence information more efficiently. In [27], MPE was selected as a feature of ultrasonic scattered echo signals to distinguish whether biological tissues have been denatured during HIFU treatment. Although MPE has the advantages of simple calculation and strong anti-noise ability, it ignores the amplitude difference between the same permutation patterns and excludes the amplitude information of the time series [28,29]. In order to overcome the inherent defects of MPE methods, Rostaghi proposed a nonlinear analysis method, named multiscale dispersion entropy (MDE) [30]. MDE has the advantages of simple calculation and strong anti-noise ability. Furthermore, MDE can analyze time series complexity including amplitude information. In [31], MDE was applied to extract the nonlinear features of ship-radiated noise. In [32], partial discharge fault diagnosis based on the MDE method was implemented. Hamed Azami employed the MDE method to analyze the complexity of biomedical time series [33]. However, as the scale factor increases, the length of the time series becomes shorter, which results in entropy fluctuation and entropy error for MDE [34,35]. The problem is caused by the traditional MDE coarse-grained process. To solve the problem, the coarse-grained process was improved. The method of improved multiscale dispersion entropy (IMDE) was proposed. The IMDE method can effectively solve the problem of entropy fluctuation and improve the stability and reliability of multiscale entropy.

In this paper, CS was used to denoise the ultrasonic scattered echo signal. The IMDE method was proposed based on traditional MDE. Considering the stability and reliability of IMDE, the IMDE method is employed to analyze the difference between normal biological tissues and denatured biological tissues. Comparing with wavelet-IMDE and EMD-IMDE methods, CS-OMP-IMDE can better identify whether biological tissues have been denatured and guide doctors to obtain a more accurate assessment of treatment effect during HIFU treatment. The outline of this paper is as follows: Section 1 is the introduction; Section 2 is the methods, which includes the methods of CS, MDE, and IMDE; Section 3 is the experimental results and analysis, which includes the experimental system, signal denoising based on CS, and comparison between MDE and IMDE; Section 4 is the discussion; Section 5 is the conclusion.

## 2. Methods

### 2.1. Compressed Sensing

Donoho proposed the compressed sensing (CS) theory, which suggests that if signals are sparse or nearly sparse, it can be reconstructed and restored to obtain denoised signals [19]. CS theory mainly includes the following three parts.

(1) Sparse expression of the signal: when most signals are not sparse in the time domain, the one-dimensional signal *X*_N*1_ should be converted into a sparse domain Ψ, which is “K-sparse” in the sparse domain Ψ (K << N). The process is

(1) f=ΨX

where *f* is the sparse representation of *X* in the sparse domain; Ψ is the sparse basis; *X* is the signal to be denoised. 

(2) Compression observation of signal: An appropriate observation matrix Φ is selected for observation and obtains the observation value *y*. The process is

(2) y=ΦΨX

where Φ is the observation matrix. In this paper, the Gaussian random matrix is selected as the observation matrix.

(3) Reconstruction of the signal: According to the *y* value in Equation (2), the denoised signal is obtained by the reconstruction method, where the reconstruction methods of L1 norm and orthogonal matching pursuit (OMP) are used for signal reconstruction, respectively. The L1 norm reconstruction method is a convex optimization algorithm, which uses the L1 norm for linear programming to achieve signal reconstruction. The OMP reconstruction method selects the atom that best matches the original signal from the observation matrix to reconstruct the sparse approximation, then, subtracts the relevant part from the observation matrix, and obtains the reconstructed signal that satisfies the sparsity after loop iteration.

### 2.2. Multiscale Dispersion Entropy

For time series $x_{n},\;n=1,2,3,\ldots ,N$, it can be mapped into $y_{n}$ using the normal cumulative distribution function.

(3) $$y_{n}=\frac{1}{\sigma \sqrt{2\pi }}\int_{-\infty }^{x_{n}}e^{\frac{-{\left(t-\mu \right)}^{2}}{2\sigma^{2}}}dt$$

where σ is the standard deviation and μ is the average value of the time series. $y_{n}$ can be mapped to the set from 1 to *c* through Equation (4).

(4) $$z_{n}^{c}=round(c\cdot y_{n}+0.5)$$

where *c* represents the number of categories. For embedding dimension m and time delay τ, the embedding vector $z_{i}^{m,c}$ can be reconstructed as

(5) $$z_{i}^{m,c}=\left\{z_{i}^{c},z_{i+d}^{c},\ldots ,z_{i+(m-1)d}^{c}\right\},i=1,2,\ldots ,N-(m-1)d$$

Through $z_{i}^{c}=v_{0},z_{i+d}^{c}=v_{1},\ldots ,z_{i+(m-1)d}^{c}=v_{m-1}$, the embedding vector $z_{i}^{m,c}$ can be mapped into the dispersion pattern $\pi_{v_{1}v_{2}\ldots v_{m-1}}$. Since each element in each $\pi_{v_{1}v_{2}\ldots v_{m-1}}$ has *c* values, the number of potential dispersion patterns is $c^{m}$. The relative frequency of each potential dispersion pattern can be defined as

(6) $$p(\pi_{v_{0}v_{1}\ldots v_{m-1}})=\frac{Number(\pi_{v_{0}v_{1}\ldots v_{m-1}})}{N-(m-1)d}$$

where $Number(\pi_{v_{1}v_{2}\ldots v_{m-1}})$ is the mapping number of the dispersion pattern $\pi_{v_{1}v_{2}\ldots v_{m-1}}$; the dispersion entropy (DE) is calculated as follows

(7) $$DE(x,m,c,d)=-\sum_{\pi =1}^{c^{m}}p(\pi_{v_{0}v_{1}\ldots v_{m-1}})\ln(p(\pi_{v_{0}v_{1}\ldots v_{m-1}}))$$

The coarse-grained time series can be expressed as

(8) $$M_{k,j}^{\left(\tau \right)}=\frac{1}{\tau }\sum_{n=(j-1)\tau +k}^{j\tau +k-1}x_{n}\;1\leq j\leq \frac{N}{\tau },1\leq k\leq \tau$$

The multiscale dispersion entropy (MDE) can be defined as

(9) $$MDE\left(x,m,c,d,\tau \right)=DE(M_{k}^{\left(\tau \right)},m,c,d)$$

### 2.3. Improved Multiscale Dispersion Entropy

**Figure 1:**
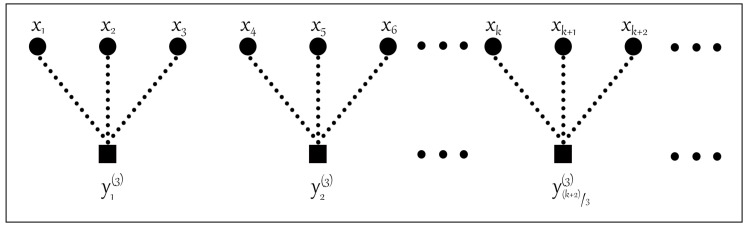
The traditional coarse-grained process for scale factor τ=3.

**Figure 2:**
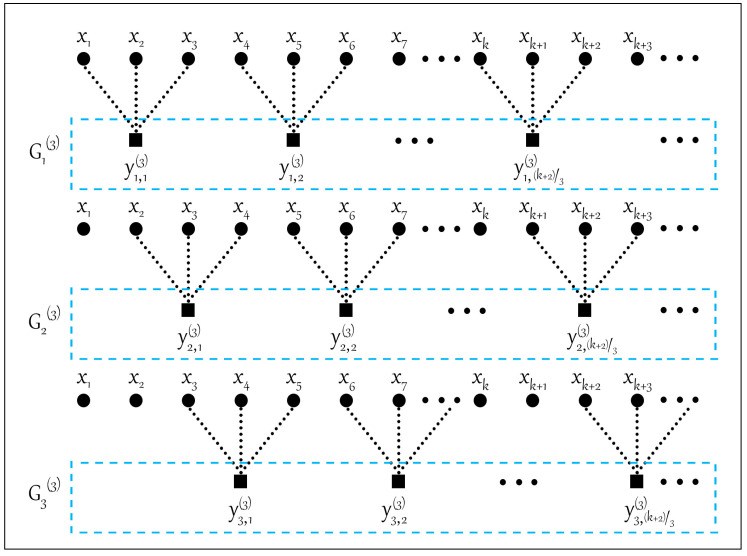
The improved coarse-grained process for scale factor *τ* = 3.

The traditional coarse-grained process is shown in Figure 1. In the traditional coarse-grained process, the number of elements in the coarse-grained time series decreases with the increase in scale factor, which will lead to instability of the entropy value. In order to improve the stability of MDE, the improved coarse-grained process is shown in Figure 2. Compared with the traditional coarse-grained process, under the same time scale τ, τ sets of time series can be obtained after the improved coarse-grained process, which can solve the unstable problem of entropy. 

For the one-dimensional time series, τ sets of new coarse-grained time series $G_{i}^{\left(\tau \right)}=\left\{y_{i,1}^{\left(\tau \right)},y_{i,2}^{\left(\tau \right)},\ldots |i=1,2,\ldots ,\tau \right\}$ can be obtained after the improved coarse-grained process, where $y_{i,j}^{\left(\tau \right)}$ is expressed as

(10) $$y_{i,j}^{\left(\tau \right)}=\frac{\sum_{f=0}^{\tau -1}x_{f+i+\tau \left(j-1\right)}}{\tau }$$

For each scale factor τ and embedded dimension d, the DE value of each time series in $G_{i}^{\left(\tau \right)}|\left(i=1,2,\ldots \tau \right)$ is calculated, respectively, and its average value of the τ sets of time sequences is defined as the improved multiscale dispersion entropy (IMDE).

(11) $$IMDE\left(x,m,c,d,\tau \right)=DE(G_{i}^{\left(\tau \right)},m,c,d)$$

### 2.4. Intraclass Distance and Interclass Distance

In feature selection, the intraclass distance and interclass distance are widely used as indicators of separability and compactness. The smaller intraclass distance and larger interclass distance mean that the features have better compactness and separability. For sample sets for each pattern in n-dimensional space {a(i)|i=1,2…,k}, the definition of intraclass distance is as follows.

(12) $$D_{\mathrm{int}ra}=\sqrt{\frac{1}{k}\sum_{j=1}^{k}\left[\frac{1}{k-1}\sum_{i=1,i\neq j}^{k}\sum_{k=1}^{n}{\left(a_{k}^{j}-a_{k}^{i}\right)}^{2}\right]}$$

The definition of interclass distance is as follows.

(13) $$D_{\mathrm{int}er}=\sqrt{\sum_{k=1}^{n}{\left(\bar{m_{1_{k}}}-\bar{m_{2_{k}}}\right)}^{2}}$$

where $\bar{m_{1_{k}}}$ and $\bar{m_{2_{k}}}$ are the mean value of the two types of pattern sample sets. 

## 3. Experimental Results and Analysis

### 3.1. Experimental System

**Figure 3:**
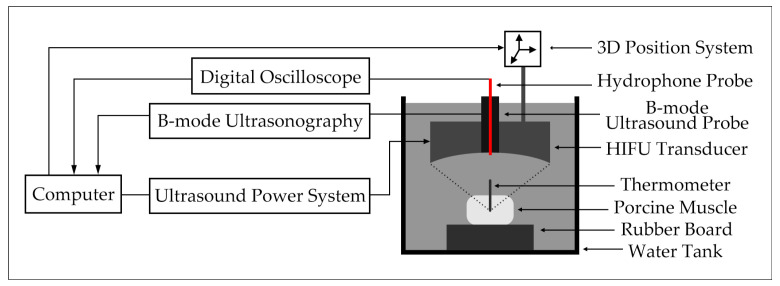
Experimental system.

**Figure 4:**
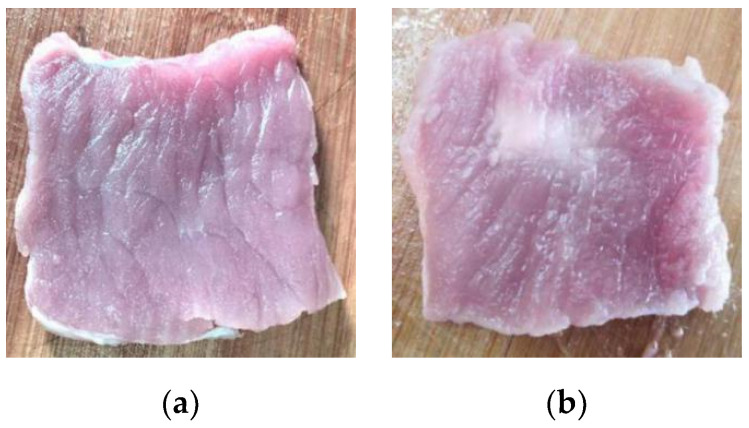
The pictures of normal and denatured tissues in vitro. (**a**) Normal tissue; (**b**) Denatured tissue.

Figure 3 shows the diagram of the HIFU experimental system. Before the HIFU experiment, povidone and 95% alcohol were mixed at a ratio of 1:4. Then, the mixed solution was mixed with water at a volume ratio of 1:20 and took one hour to remove oxygen from the water to obtain oxygen-free water. The sizes of the porcine muscle tissue samples in vitro were prepared as 60 × 55 × 50 mm. Then, all the porcine muscle tissue samples in vitro were degassed to prevent it affecting the experimental results. The target region of the fresh porcine muscle tissues in vitro was irradiated by a HIFU transducer (PRO2008, Shenzhen, China) with a center frequency of 1.39 MHz to change the biological tissue characteristics. At the same time, the temperature of the HIFU-irradiated porcine muscle target region was measured by a thermometer. After turning off the HIFU ultrasound probe, B-mode ultrasonography (SSI-5500, SonoScape, Shenzhen, China) was used to monitor the treatment process of HIFU and the sampling frequency of the ultrasound probe was 20 MHz. A fiber optic hydrophone was used to obtain the B-mode ultrasonic scattered echo signal. Then, the signal was converted into a digital signal by a digital oscilloscope and saved in the PC. In this HIFU experiment, 300 ultrasonic scattered echo signals of normal and denatured tissues were collected (including 150 cases of normal tissues and 150 cases of denatured tissues). Figure 4 shows the pictures of normal tissue and denatured tissue in vitro.

### 3.2. Signal Denoising Based on CS

The simulated signal was analyzed by compressed sensing (CS). The simulated signal was as follows.

y=2cos(200000(π/256t))+sin(200000(π/128t))

**Figure 5:**
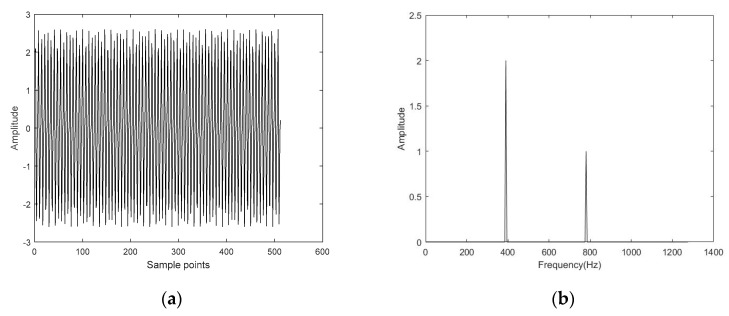
Time-domain diagram and frequency spectrum of the simulated signal. (**a**) Time-domain diagram; (**b**) Frequency spectrum.

**Figure 6:**
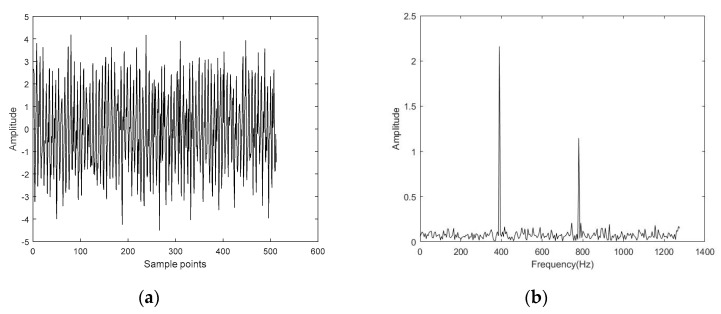
Time-domain diagram and frequency spectrum of the noisy simulated signal with signal-to-noise ratio of 5 dB. (**a**) Time-domain diagram; (**b**) Frequency spectrum.

**Figure 7:**
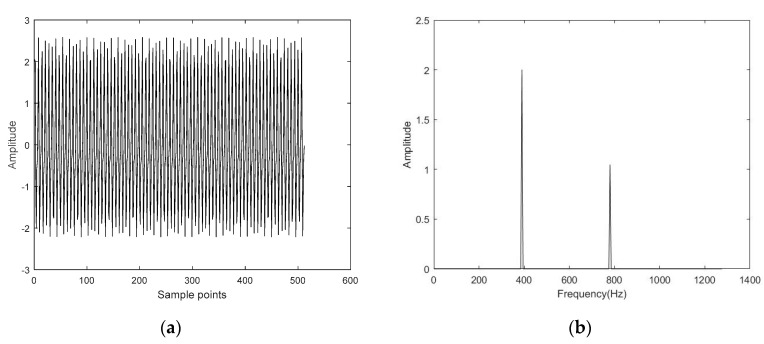
Time-domain diagram and frequency spectrum of the denoised signal by the reconstruction method of OMP. (**a**) Time-domain diagram; (**b**) Frequency spectrum.

**Figure 8:**
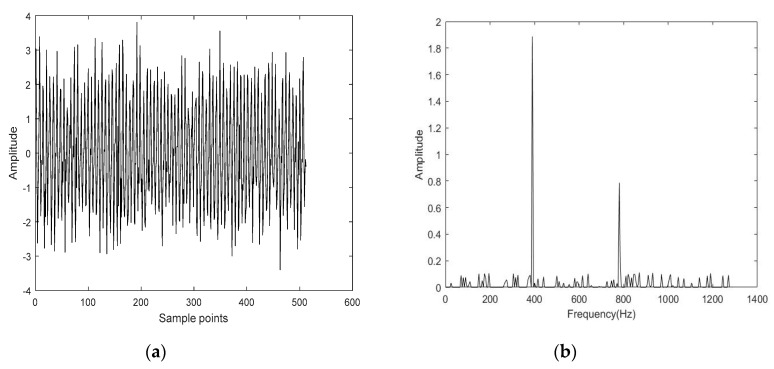
Time-domain diagram and frequency spectrum of the denoised signal by the reconstruction method of L1 Norm. (**a**) Time-domain diagram; (**b**) Frequency spectrum.

The time-domain waveform and frequency spectrum of the simulated signal are shown in Figure 5. Then, Gaussian white noise was added to the simulated signal so that the signal-to-noise ratio of the noisy simulated signal was 5 dB. The time-domain diagram and frequency spectrum of the noisy simulated signal are shown in Figure 6. The noisy simulated signal was analyzed by CS. The methods of L1 norm and OMP were employed to reconstruct and obtain the denoised signals, respectively. The time-domain waveform and frequency spectrum of the denoised signals are shown in Figure 7 and Figure 8. It can be observed that the time-domain waveform and frequency spectrum of the denoised signal obtained by the OMP reconstruction method was closer to that of the simulated signal. The denoised signal obtained by the L1 norm reconstruction method contained more noise components. The denoised effect of the OMP reconstruction was obviously better than that of the L1 norm reconstruction. 

Meanwhile, we compared with the traditional wavelet and empirical mode decomposition (EMD) denoising methods. Table 1 shows the comparison results of various denoising methods on the noisy simulated signals with different signal-to-noise ratios (SNR). The results show that the signal-to-noise ratio of the CS-OMP method is the highest and the mean square error (MSE) is the lowest compared with wavelet and EMD denoising methods, which means that the CS-OMP method can obtain a better denoising effect.

**Figure 9:**
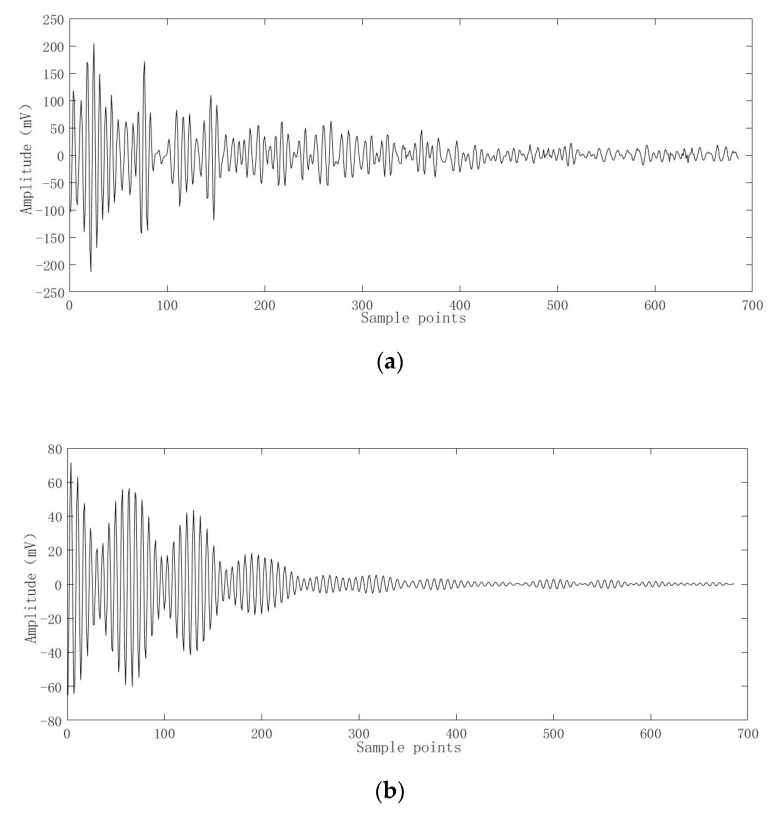
Ultrasonic scattered echo signals before and after denoising by the CS-OMP method. (**a**) Ultrasonic scattered echo signals before denoising; (**b**) Ultrasonic scattered echo signals after denoising.

The CS-OMP method was used to denoise the actual ultrasonic scattered echo signal. The waveform of the ultrasonic scattered echo signal before and after denoising is shown in Figure 9. It can be clearly seen that the denoised signal has obvious pulse waveform, and the waveform shows oscillation attenuation.

### 3.3. Comparison between MDE and IMDE of Simulated Signal

**Figure 10:**
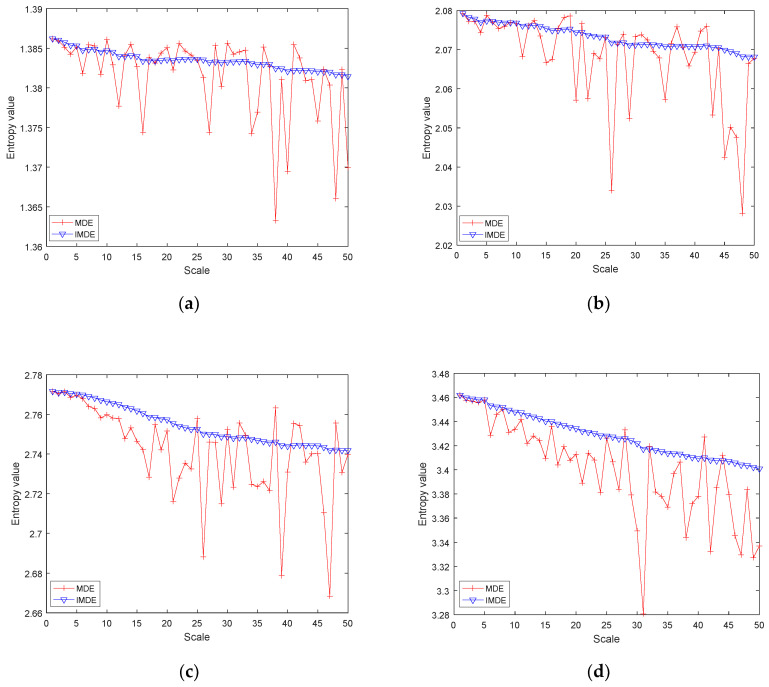
Different entropies values of the simulated signal using different embedding dimension. (**a**) m = 4; (**b**) m = 5; (**c**) m = 6; (**d**) m = 7.

To illustrate the advantages of the IMDE method, white Gaussian noise of 5000 data points was generated as the simulated signal. According to the published reports [30,33], we chose the number of categories as 2 and the delay time as 2. The MDE and IMDE of the simulated signal were calculated when the embedding dimension was 4, 5, 6, and 7, respectively. The results are shown in Figure 10. It can be clearly seen from Figure 10 that both MDE and IMDE show an overall downward trend with the increase in scale factors. However, the value of MDE fluctuates significantly with the increase in scale factor, which reduces the stability of multiscale entropy. The value of IMDE fluctuates less and has better stability, which means that the improvement in the coarse-grained process is effective.

At the same time, we also analyzed the standard deviation of MDE and IMDE of simulated signals at different data points. The standard deviations of MDE and IMDE of simulated signals at 500, 1000, 3000, and 5000 data points are shown in Table 2. It can be clearly seen from Table 2 that the standard deviation of entropy is smaller as the number of data points increases. In addition, the standard deviation of IMDE for simulated signals with different data points is significantly smaller than MDE, which indicates that IMDE has better stability in analyzing the complexity of time series than MDE.

### 3.4. MDE and IMDE of Actual Ultrasonic Scattered Echo Signals

**Figure 11:**
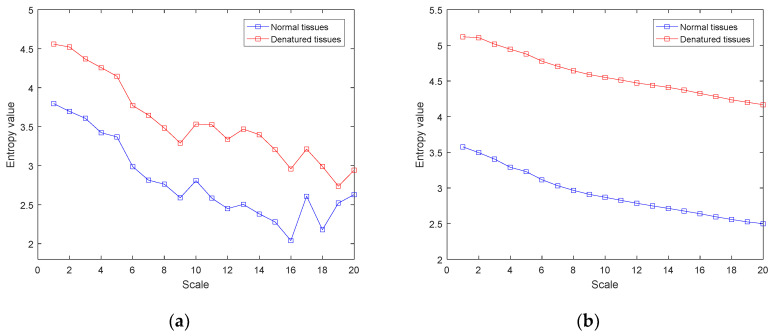
The results of MDE and IMDE of ultrasonic scattered echo signals. (**a**) MDE; (**b**) IMDE.

The MDE and IMDE methods were used to calculate the entropy value of the ultrasonic scattered echo signals after CS-OMP denoising. The results are shown in Figure 11. It can be clearly seen from Figure 11 that the MDE and IMDE values of the ultrasonic scattered echo signals of the denatured tissue are higher than that of the normal tissues. Both MDE and IMDE can distinguish normal tissues and denatured tissues in vitro. However, compared with the MDE, the IMDE curve of ultrasonic scattered echo signals is smoother and more stable. The difference in IMDE between normal and denatured tissues is more obvious at different scales.

**Figure 12:**
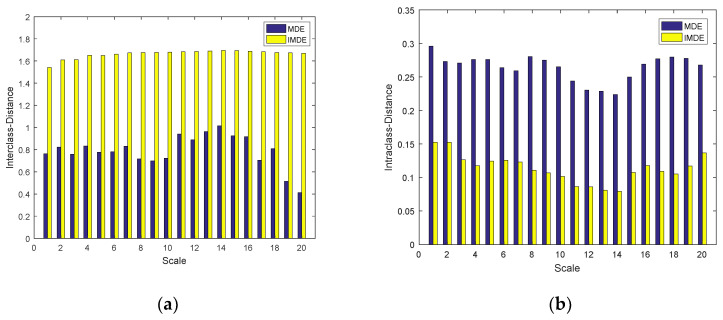
The interclass distance and intraclass distance of MDE and IMDE within different scale factors. (**a**) Interclass distance of MDE and IMDE within different scale factors; (**b**) Intraclass distance of MDE and IMDE within different scale factors.

In order to further prove the advantage of IMDE, the two indicators of interclass distance and intraclass distance were used to measure the separability and compactness of the MDE and IMDE of the normal and denatured tissues in vitro. Figure 12 shows the interclass distance and intraclass distance of MDE and IMDE within scale factors 1–20. From Figure 12, it can be clearly observed that the interclass distance of IMDE is greater than MDE and the intraclass distance of IMDE is less than MDE. This indicates that IMDE has better separability and compactness to identify whether biological tissues have been denatured compared with MDE. At the same time, IMDE has the maximum interclass distance and the minimum interclass distance when the scale factor is 14.

## 4. Discussion

In HIFU treatment, the ultrasonic scattered echo signal is often used to identify whether the biological tissues have been denatured. In this process, the ultrasonic echo signal contains a large number of noises that affect the identification results, and it is necessary to find a method that can accurately reflect the characteristics of denatured tissue.

In this paper, the CS-OMP method is applied to signal denoising. Compared with the traditional wavelet and EMD methods, the CS-OMP method can obtain a better denoising effect (Figure 8, Table 1). In addition, the proposed IMDE method can effectively improve the stability of MDE (Figure 10, Table 2). IMDE can also better distinguish normal and denatured tissues (Figure 11 and Figure 12). Meanwhile, the various identification methods of denatured biological tissue including CS-OMP-IMDE, wavelet-IMDE and EMD-IMDE are used to distinguish normal tissue and denatured tissue. The identification effect shows that the interclass distance and the intraclass distance of the CS-OMP-IMDE method are 1.6964 and 0.0794; the interclass distance and the intraclass distance of the wavelet-IMDE recognition method are 1.2924 and 0.1075; the interclass distance and the intraclass distance of the EMD-IMDE method are 1.1537 and 0.1203. This proves the advantage of the CS-OMP-IMDE method in the identification of denatured biological tissue. These above results support the hypothesis that the combination of the CS-OMP and IMDE methods is able to denoise and identify. We can obtain better identification of denatured tissue. In future work, entropy imaging may be adopted to detect the target region of HIFU.

## 5. Conclusions

This paper realizes the identification of denatured tissues based on CS and IMDE of the ultrasonic scattered echo signal. In the analysis of CS, the CS-OMP method can denoise the ultrasonic scattered echo signal effectively. In view of the shortcomings of the traditional MDE method, the IMDE method is proposed by improving the coarse-grained process of MDE. The proposed IMDE has better stability compared with MDE. MDE and IMDE are applied to ultrasonic scattered echo signals of normal and denatured tissues. The results show that the interclass distance of IMDE is greater than MDE, and the intraclass distance of IMDE is less than MDE. IMDE has better compactness and separability to identify whether biological tissues have been denatured. Furthermore, when the scale factor is 14, IMDE can obtain the optimal identification effect for normal tissues and denatured tissues during HIFU treatment. Comparing with the wavelet-IMDE and EMD-IMDE methods, CS-OMP-IMDE has better effect in the identification of denatured biological tissue.

## Figures and Tables

**Table 1:** The comparison of effects with different denoising methods.

SNR of Noisy Simulated Signals	Denoising Methods	SNR/dB	MSE
	CS-OMP	13.2699	0.3431
2 dB	Wavelet	3.8226	1.0182
	EMD	2.9746	1.2596
	CS-OMP	16.5219	0.2360
5 dB	Wavelet	5.1166	0.8773
	EMD	5.7382	0.8167
	CS-OMP	31.6592	0.0413
10 dB	Wavelet	4.9736	0.8919
	EMD	9.7579	0.5141

**Table 2:** Standard deviation of the MDE and IMDE of the simulated signal at different data points.

Entropy	Number of Data Points			
	500	1000	3000	5000
MDE	0.5481	0.3134	0.0974	0.0576
IMDE	0.2787	0.1402	0.0354	0.0227

## References

[1] Feijoo E.R.C., Sivaraman A., Barret E., Sanchez-Salas R., Galiano M., Rozet F., Prapotnich D., Cathala N., Mombet A., Cathelineau X. (2016). Focal High-intensity Focused Ultrasound Targeted Hemiablation for Unilateral Prostate Cancer: A Prospective Evaluation of Oncologic and Functional Outcomes. Eur. Urol..

[2] Lang B.H.H., Woo Y.-C., Wong C.K.H. (2017). High-Intensity Focused Ultrasound for Treatment of Symptomatic Benign Thyroid Nodules: A Prospective Study. Radiology.

[3] You Y., Wang Z., Ran H., Zheng Y., Wang N., Xu J., Wang Z., Chen Y., Li P. (2016). Nanoparticle-enhanced synergistic HIFU ablation and transarterial chemoembolization for efficient cancer therapy. Nanoscale.

[4] Bailey M.R., Khokhlova V.A., Sapozhnikov O.A., Kargl S.G., Crum L.A. (2003). Physical mechanisms of the therapeutic effect of ultrasound (a review). Acoust. Phys..

[5] Yin N., Hu L., Xiao Z.-B., Liu C., Chen W.-Z., Roberts N., Chen J., Wang Z.-B. (2018). Factors influencing thermal injury to skin and abdominal wall structures in HIFU ablation of uterine fibroids. Int. J. Hyperth..

[6] Weiss N., Goldberg S.N., Sosna J., Azhari H. (2014). Temperature-density hysteresis in X-ray CT during HIFU thermal ablation: Heating and cooling phantom study. Int. J. Hyperth..

[7] Weiss N., Sosna J., Goldberg S.N., Azhari H. (2014). Non-invasive temperature monitoring and hyperthermic injury onset detection using X-ray CT during HIFU thermal treatment in ex vivo fatty tissue. Int. J. Hyperth..

[8] Rouvière O., Girouin N., Glas L., Ben Cheikh A., Gelet A., Mège-Lechevallier F., Rabilloud M., Chapelon J.-Y., Lyonnet D. (2010). Prostate cancer transrectal HIFU ablation: Detection of local recurrences using T2-weighted and dynamic contrast-enhanced MRI. Eur. Radiol..

[9] Quesson B., Laurent C., Maclair G., De Senneville B.D., Mougenot C., Ries M., Carteret T., Rullier A., Moonen C.T. (2011). Real-time volumetric MRI thermometry of focused ultrasound ablation in vivo: A feasibility study in pig liver and kidney. NMR Biomed..

[10] Yang C., Zhu H., Wu S., Bai Y., Gao H. (2010). Correlations between B-mode ultrasonic image texture features and tissue temperature in microwave ablation. J. Ultrasound Med..

[11] Wust P., Cho C.H., Hildebrandt B., Gellermann J. (2006). Thermal monitoring: Invasive, minimal-invasive and non-invasive approaches. Int. J. Hyperth..

[12] Seip R., Tavakkoli J., Carlson R.F., Wunderlich A. High-intensity focused ultrasound(HIFU) multiple lesion imagine: Comparison of detection algorithms for real-time treatment control. Proceedings of the 2002 IEEE Ultrasonics Symposium.

[13] Paris M.T., Bell K.E., Avrutin E., Mourtzakis M. (2020). Ultrasound image resolution influences analysis of skeletal muscle composition. Clin. Physiol. Funct. Imaging.

[14] Lewis M.A., Staruch R.M., Chopra R. (2015). Thermometry and ablation monitoring with ultrasound. Int. J. Hyperth..

[15] Zhou Z., Wu W., Wu S., Xia J., Wang C.-Y., Yang C., Lin C.-C., Tsui P.-H. (2014). A survey of ultrasound elastography approaches to percutaneous ablation monitoring. Proc. Inst. Mech. Eng. Part H J. Eng. Med..

[16] Zhou Z., Tai D.-I., Wan Y.-L., Tseng J.-H., Lin Y.-R., Wu S., Yang K.-C., Liao Y.-Y., Yeh C.-K., Tsui P.-H. (2018). Hepatic Steatosis Assessment with Ultrasound Small-Window Entropy Imaging. Ultrasound Med. Biol..

[17] Huang N.E., Shen Z., Long S.R., Wu M.C., Shih H.H., Zheng Q., Yen N.-C., Tung C.C., Liu H.H. (1998). The empirical mode decomposition and the Hilbert spectrum for nonlinear and non-stationary time series analysis. Proc. R. Soc. A Math. Phys. Eng. Sci..

[18] Murashige A., Hiro T., Fujii T., Imoto K., Murata T., Fukumoto Y., Matsuzaki M. (2005). Detection of Lipid-Laden Atherosclerotic Plaque by Wavelet Analysis of Radiofrequency Intravascular Ultrasound Signals: In vitro validation and preliminary in vivo application. J. Am. Coll. Cardiol..

[19] Dong B., Mao Y., Osher S., Yin W. (2010). Fast linearized Bregman iteration for compressive sensing and sparse denoising. Commun. Math. Sci..

[20] Bai L., Maechler P., Muehlberghuber M., Kaeslin H. High-speed compressed sensing reconstruction on FPGA using OMP and AMP. Proceedings of the 2012 19th IEEE International Conference on Electronics, Circuits, and Systems (ICECS 2012).

[21] Pichardo S., Sin V.W., Hynynen K. (2010). Multi-frequency characterization of the speed of sound and attenuation coefficient for longitudinal transmission of freshly excised human skulls. Phys. Med. Biol..

[22] Zderic V., Keshavarzi A., Andrew M.A., Vaezy S., Martin R.W. (2004). Attenuation of porcine tissues in vivo after high-intensity ultrasound treatment. Ultrasound Med. Biol..

[23] Seip R., Ebbini E.S., O’Donnell M.B., Cain C. Non-invasive detection of thermal effects due to highly focused ultrasonic fields. Proceedings of the 1993 IEEE Ultrasonics Symposium.

[24] Suomi V., Han Y., Konofagou E., Cleveland R.O. (2016). The effect of temperature dependent tissue parameters on acoustic radiation force induced displacements. Phys. Med. Biol..

[25] Mobasheri S., Behnam H., Rangraz P., Tavakkoli J. (2016). Radio Frequency Ultrasound Time Series Signal Analysis to Evaluate High-intensity Focused Ultrasound Lesion Formation Status in Tissue. J. Med. Signals Sensors.

[26] Tsui P.-H., Wan Y.-L. (2016). Effects of Fatty Infiltration of the Liver on the Shannon Entropy of Ultrasound Backscattered Signals. Entropy.

[27] Liu B., Hu W.P., Zou X., Ding Y.J., Qian S.Y. (2019). Recognition of denatured biological tissue based on variational mode decomposition and multi-scale permutation entropy. Acta Phys. Sin..

[28] Fadlallah B., Chen B., Keil A., Principe J. (2013). Weighted-permutation entropy: A complexity measure for time series incorporating amplitude information. Phys. Rev. E.

[29] Wu S.-D., Wu C.-W., Lin S.-G., Wang C.-C., Lee K.-Y. (2013). Time Series Analysis Using Composite Multiscale Entropy. Entropy.

[30] Rostaghi M., Azami H. (2016). Dispersion Entropy: A Measure for Time-Series Analysis. IEEE Signal Process. Lett..

[31] Li Z., Li Y., Zhang K., Guo J. (2019). A Novel Improved Feature Extraction Technique for Ship-Radiated Noise Based on IITD and MDE. Entropy.

[32] Shang H., Li F., Wu Y. (2019). Partial Discharge Fault Diagnosis Based on Multi-Scale Dispersion Entropy and a Hypersphere Multiclass Support Vector Machine. Entropy.

[33] Azami H., Fernández A., Escudero J. (2019). Multivariate Multiscale Dispersion Entropy of Biomedical Times Series. Entropy.

[34] Azami H., Rostaghi M., Abásolo D., Escudero J. (2017). Refined Composite Multiscale Dispersion Entropy and its Application to Biomedical Signals. IEEE Trans. Biomed. Eng..

[35] Yan X., Jia M. (2019). Intelligent fault diagnosis of rotating machinery using improved multiscale dispersion entropy and mRMR feature selection. Knowl. Based Syst..

